# Programmable Stapling Peptide Based on Sulfonium as Universal Vaccine Adjuvants for Multiple Types of Vaccines

**DOI:** 10.1002/advs.202409567

**Published:** 2025-01-29

**Authors:** Yaping Zhang, Chenshan Lian, Wenlong Lai, Leying Jiang, Yun Xing, Huiting Liang, Jin Li, Xinming Zhang, Jianhui Gan, Zigang Li, Feng Yin

**Affiliations:** ^1^ State Key Laboratory of Chemical Oncogenomics School of Chemical Biology and Biotechnology Peking University Shenzhen Graduate School Shenzhen 518055 P. R. China; ^2^ Pingshan Translational Medicine Center Shenzhen Bay Laboratory Shenzhen 518118 P. R. China; ^3^ Shenzhen Kangtai Biological Products Co. Ltd. Shenzhen 518057 P. R. China; ^4^ Beijing Minhai Biotechnology Co. Ltd. Beijing 102609 P. R. China

**Keywords:** nano‐vaccine, stapling peptides, sulfonium center, universal adjuvant

## Abstract

Adjuvants are non‐specific immune enhancers commonly used to improve the responsiveness and persistence of the immune system toward antigens. However, due to the undefined chemical structure, toxicity, non‐biodegradability, and lack of design technology in many existing adjuvants, it remains difficult to achieve substantive breakthroughs in the adjuvant research field. Here, a novel adjuvant development strategy based on stapling peptides is reported to overcome this challenge. The nano‐vaccine incorporating peptide adjuvant and recombinant HBsAg protein not only induced strong antibody titers that are equivalent to aluminum adjuvanted vaccines but also simultaneously activated T‐cell immune response. Similar results are also observed in herpes zoster vaccine and more complex influenza vaccine. The mechanism analysis demonstrates that antigen is efficiently carried into antigen‐presenting cells (APCs) by peptide, further promoting the secretion of cytokines and activation of APCs. In addition, by redesigning the adjuvant, it is found that the sulfonium centers, rather than the sequence of peptide played an important role in immune activation. This discovery may provide a new paradigm for the rational design of peptide‐based adjuvants. In brief, this study demonstrates that stapling peptides with sulfonium centers can provide a well‐defined, programmable, biocompatible, and effective adjuvant for multiple types of vaccines.

## Graphical abstract

A stapling peptide with two sulfonium centers is developed to be used as an excellent vaccine adjuvant candidate for the Hepatitis B vaccine, influenza vaccine, and herpes zoster vaccine. Just by changing the amino acids and ring types, it is easy to program peptide adjuvants to adapt to multiple vaccine subtypes and synergistically induce humoral and cellular immune activation.

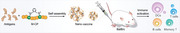

## 1 Introduction

Vaccines are effective tools for preventing respiratory diseases, and their role in tumor immunotherapy has gradually become prominent.^[^ ^1^ ^]^ Subunit vaccines and split vaccines, to some extent, avoided the safety issues of whole‐pathogen vaccines and hold great potential in clinical application.^[^ ^2^ ^]^ While non‐adjuvanted subunit vaccines can only lead to limited immune efficacy.^[^ ^3^ ^]^ Adjuvant, usually as a constituent, was added to vaccines to enhance the immune intensity and persistence. At present, aluminum is the most widely used adjuvant in commercialized vaccines like Hepatitis B and Pertussis vaccine. However, aluminum's poor cellular immunity makes it unsuitable for all vaccines.^[^ ^4^ ^]^ Recent studies showed that powerful adjuvants, such as oil in water mixture (MF59, AS01, AS03, AF03, and Matrix‐M) are effective in stimulating a strong immune response.^[^ ^4^, ^5^ ^]^ Currently, natural derivative components in most complex adjuvants are poorly chemically defined and difficult to characterize and standardize, posing another challenge to the safety application of novel adjuvants.^[^ ^2^ ^]^ Moreover, the current adjuvant mostly stems from experience accumulation. The rapid development of various types of vaccines has put forward higher requirements for the rational design of new vaccine adjuvants. Thus, a programmable adjuvant with a chemically defined structure, good biosafety, easily mass‐manufactured, and adaptability to various vaccine subtypes, is urgently needed.

Biocompatible peptides provide potential candidates for novel vaccine adjuvants. For example, the self‐assembled peptides (Q11 and RADA16) and truncated peptides derived from pathogenic microorganisms or self‐adjuvanted protein (glucosamine muramyl dipeptide, β‐defensin, and Hp91 from high mobility group box1) have proven to be excellent adjuvant candidates.^[^ ^6^ ^]^ However, due to the low stability and membrane permeability of peptides, there is still a long way to go for the clinical application of linear peptide adjuvant.^[^ ^7^ ^]^ At present, various peptide cyclization strategies have been applied to improve the pharmacological properties of peptide drugs.^[^ ^8^ ^]^ On the one hand, cyclization modification increases the stability and half‐life of peptides.^[^ ^8^, ^9^ ^]^ On the other hand, the abundant chemical modification sites of peptides provide more possibilities for the rational design of various peptide‐based adjuvants. Thus, utilizing peptide stabilization modification strategies in the design of peptide‐based adjuvants is expected to offer new options for the development of vaccine adjuvants.

In this work, we proposed a novel vaccine adjuvant development strategy based on sulfonium stapling peptides with only 9 amino acids for the first time and verified its effect as an immune enhancer in several common vaccines. In contrast to natural extract in other approved vaccine adjuvants, linear peptide material was synthesized by the classical solid‐phase peptide synthesis method and cyclized at two methionine positions according to our previous report.^[^ ^7b^ ^]^ Then the effectiveness of the peptide vaccine adjuvant (named M‐CP) was evaluated in two recombinant protein vaccines (hepatitis B and herpes zoster vaccine) and one lysis vaccine (the influenza vaccine). Our study revealed that these vaccines supplemented with M‐CP could induce strong and effective humoral and cellular immune responses. In addition, M‐CP has demonstrated its unique advantages in terms of long‐term effectiveness and biosafety. Further utilizing the modifiability of peptide, we conducted a detailed analysis of the action mechanisms of the M‐CP peptide adjuvant, showing the important role of sulfonium centers in antigen delivery and performance enhancement. This discovery is expected to provide new directions for redesigning peptide‐based adjuvants and the construction of stapling peptide‐based adjuvant libraries in the future. We also believe that these novel vaccine adjuvants utilizing stable peptide modification strategies will contribute to the current and future arrangements for the prevention and control of respiratory virus diseases and to the development of the vaccine industry.

## 2 Results and Discussion

### 2.1 Sulfonium‐based Stapling Peptide is an Excellent Candidate as Hepatitis B Vaccine Adjuvant

**Figure 1:**
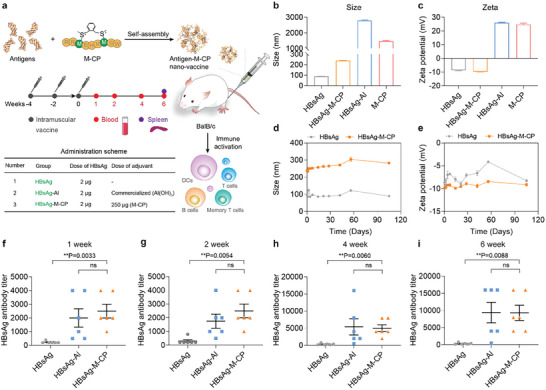
Characterization of M‐CP as hepatitis B vaccine adjuvant. a) Schematic illustration of the sulfonium‐based stapling peptide as a vaccine adjuvant to stimulate cellular and humoral immunity. b,c) DLS (Dynamic light scattering) analysis on the b) particle size and c) zeta potential of the nano‐vaccine (HBsAg‐M‐CP) and commercial hepatitis B vaccine (HBsAg‐Al). d,e) The change of the d) particle size and e) zeta potential of the nano‐vaccine over time at 4 °C. f–i) The anti‐HBsAg antibody titer at 1, 2, 4, and 6 weeks after the last immunization with indicated vaccines (n = 6). The data were presented as mean ± SEM and analyzed by one‐way ANOVA with Turkey multiple comparisons post‐test (^*^ *p* < 0.05, ^**^ *p* < 0.01, ^***^ *p* < 0.001, ^****^ *p* < 0.0001).

Our recent study on the sulfonium‐based stapling peptide system in neoantigen delivery demonstrated that solely peptide carrier can induce non‐specific anti‐tumor therapeutic effects, which manifested as moderate inhibition of tumor metastasis and growth.^[^ ^10^ ^]^ We speculated that sulfonium‐based stapling peptide might exert its adjuvant properties in immunotherapy. As previously pointed out, cationic liposomes and PEI with strong positive charges could act as vaccine adjuvants by activating the non‐specific immune responses.^[^ ^11^ ^]^ At present, many inorganic cationic salts have been used as adjuvants for recombinant protein antigen‐based vaccines, such as the classic aluminum adjuvant (Al^3+^), and the emerging Mn^2+^ adjuvant.^[^ ^12^ ^]^ From the sequence perspective (Figure S1, Supporting Information), the amino acid sequences and two sulfonium centers induced by cyclization gave relatively strong positive charge, which indicated the potential vaccine adjuvant properties of M‐CP. Inspired by the positively charged chemical structure and non‐specific immune enhancement effects of this sulfonium‐based stapling peptide carrier, we incorporated it (Figure S1, Supporting Information) into multiple vaccines as a novel adjuvant. For proof of concept, HBsAg (Hepatitis B surface antigen), one of the most widely used vaccine composition, was mixed with M‐CP (the mixture named HBsAg‐M‐CP). Then the mixture was characterized by dynamic light scattering (DLS) and scanning electron microscope (SEM). Here, the hepatitis B vaccine with an aluminum adjuvant was used as a positive control (named HBsAg‐Al). As shown in **Figures** **1**a–c and S2 (Supporting Information), the positively charged M‐CP induced the assembly of the regular nanospheres with HBsAg. When the concentration of M‐CP reached 250 µg mL^−1^, the addition of M‐CP barely affected the negative charge of the system with HBsAg and was chosen for subsequent studies (Figure S2a–c, Supporting Information). Different from HBsAg‐Al, the nanoparticles of HBsAg‐M‐CP were uniformly distributed with a particle size of 239 ± 4.3 nm, with a polydispersity index (PDI) <0.1 and a zeta potential at −9.8 ± 0.3 mV (Figure 1b,c). In addition, this mixture could be stored stably at 4 °C for >105 days, without significant changes in particle size and zeta potential, showing potential applications of M‐CP as a vaccine adjuvant in the future (Figure 1d,e).

To further evaluate the effectiveness of M‐CP as a vaccine adjuvant, each Balb/c mice were treated with the mixture of recombinant HBsAg protein (2 µg) and M‐CP (250 µg) three times at 14‐day intervals (Figure 1a). The serums of immunized mice were collected at 1, 2, 4, and 6 weeks and analyzed by ELISA (Figure 1a). The results revealed that without the adjuvant, HBsAg could hardly elicit anti‐HBsAg antibody response, manifested with the lowest anti‐HBsAg antibody titer during the 6‐week long monitoring (Figure 1f–i). While, the introduction of M‐CP adjuvant significantly improved the immune response of the recombinant protein, showing the antibody titers 10.7, 8.3, 13.6, and 25.5 times higher than that in HBsAg group at 1, 2, 4, and 6 weeks (Figure 1f–i), respectively. Moreover, as the concentration of M‐CP increases, the degree of the anti‐HBsAg antibody response gradually increases (Figure S3a, Supporting Information). When the dosage of M‐CP reached 250 µg, the antibody titer levels induced by the HBsAg‐M‐CP group were close to those of the HBsAg‐Al group (Figures 1f–i and S3a, Supporting Information). These results demonstrated that M‐CP could be an excellent adjuvant candidate, comparable to the classic aluminum adjuvant for subunit vaccines.

### 2.2 Candidate Peptide Adjuvant Simultaneously Induces Strong Humoral and Cellular Immune Responses

**Figure 2:**
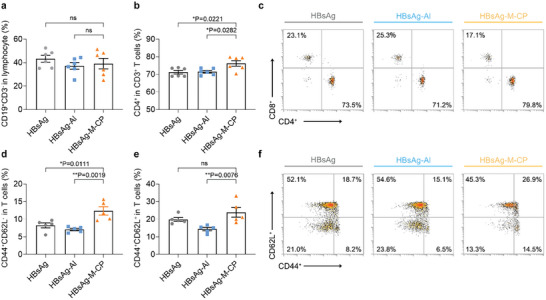
The HBsAg‐M‐CP vaccine induced strong humoral and cellular immune responses. a,b) The percentage of a) CD19^+^ B cells and b) CD3^+^CD4^+^ T cells in PBMCs at week 6. (n = 6). c) The representative scatter plots of T cells in PBMCs at week 6 from the flow cytometry analysis. d,e) Proportion of the effector memory T cells (d, CD3^+^CD44^+^CD62L^−^) and central memory T cells (e, CD3^+^CD44^+^CD62L^+^) in splenocytes after re‐stimulation with HBsAg protein. (n = 5). f) The representative scatter plots of memory T cells in splenocytes after re‐stimulation with HBsAg protein from the flow cytometry analysis. The data were presented as mean ± SEM (n ≥ 3) and analyzed by one‐way ANOVA with Turkey multiple comparisons post‐test (^*^ *p* < 0.05, ^**^ *p* < 0.01, ^***^ *p* < 0.001, ^****^ *p* < 0.0001).

The development of new‐type adjuvants usually requires consideration of the synergistic effects of cellular immunity and humoral immune activation.^[^ ^13^ ^]^ To further evaluate the activation of immune response induced by M‐CP on the cellular level, the proportion of B cells and T cells in peripheral blood at week 6 were analyzed by flow cytometry (Figure S3b–d, Supporting Information). As shown in **Figure** **2**a, there was no significant difference in the frequency of CD19^+^CD3^−^ B cells in peripheral blood mononuclear cells (PBMCs) between the HBsAg‐M‐CP group and the HBsAg Al group. This result was consistent with above antibody titer detection. While, unlike B cells, an increase in the proportion of CD4^+^ T cells was observed in the M‐CP‐containing treatment group (Figure 2b). After three immunizations, the solely HBsAg and HBsAg‐Al vaccines groups display 70% and 71% CD4^+^ T cells (Figure 2b,c), respectively. In contrast, HBsAg‐M‐CP elicited ≈75% CD4^+^ T cells (Figure 2b,c), showing the potential of M‐CP peptide adjuvants in activating the cellular immune response. In addition, this conclusion can also be supported by the significant higher proportion of memory T cells of mouse spleen cells in the HBsAg‐M‐CP group (Figure 2d–f).

We further investigated the capability of M‐CP adjuvant in inducing long‐term effectiveness. After three immunizations with the indicated formulations, the spleen single cell suspension was collected at 6 week and re‐stimulated with HBsAg protein. The HBsAg‐M‐CP group presented with the highest cell proliferation rate (Figure S3e, Supporting Information). Further analysis revealed that HBsAg‐M‐CP‐treated mice induced the maximum proportion of CD44^+^CD62L^+^CD3^+^ T cells (central memory T cells) and CD44^+^CD62L^−^CD3^+^ T cells (effector memory T cells), showing 1.4 and 1.5 times higher than the HBsAg‐Al immunized group (Figure 2d–f), respectively. The addition of aluminum adjuvant did not show any benefits in cellular immune response, presented with a similar level of immune memory cell proportion in HBsAg‐Al and HBsAg groups. These results were consistent with previous reports that aluminum adjuvants are not sufficient to induce strong cellular immunity.^[^ ^12c^ ^]^ This is also one of the key limiting factors for its application in the treatment of hepatitis B. Considering the M‐CP adjuvant's ability to induce the robust CD4^+^ T cells in PBMCs, we strongly speculate that HBsAg‐M‐CP could be an excellent vaccine candidate for Hepatitis B therapy.

### 2.3 Candidate Peptide Adjuvant is Suitable for Multiple Types of Vaccines

**Figure 3:**
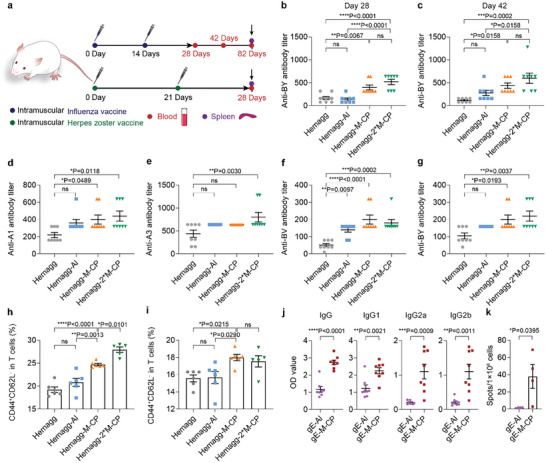
The efficacy evaluation of M‐CP as a universal adjuvant for influenza vaccine and herpes zoster vaccine. a) Schematic illustration of the vaccination plan to evaluate the universality of M‐CP for different vaccines. Balb/c mice were intramuscularly injected with different formulations as planned. b,c) The anti‐BY antibody titer at b) day 28 and c) day 42 (n = 8). d–g) The antibody titers of four hemagglutinin at day 82 (n = 8). A1, A3, BV, and BY represent influenza A virus H1N1, influenza A virus H3N2, influenza B virus Victoria, and influenza B virus Yamagata, respectively. 2*M‐CP represents double the dose of M‐CP adjuvant during immunizations. h,i) Proportion of the effector memory T cells (h, CD3^+^CD44^+^CD62L^−^) and central memory T cells ((i) CD3^+^CD44^+^CD62L^+^) in splenocytes after re‐stimulation with tetravalent influenza lysis at day 82 (n = 6). j) The OD value of IgG, IgG1, IgG2a, and IgG2b against the recombinant antigen glycoprotein E (gE) analyzed by ELISA (n = 8). k) ELISpot analysis of INF‐γ spot‐forming cells among 1 × 10^6^ splenocytes after re‐stimulation with gE protein at day 28 (n = 4). The data were presented as mean ± SEM (n ≥ 3). The multi‐component comparisons data b–i) were analyzed by one‐way ANOVA with Turkey multiple comparisons post‐test. The two‐component data j,k) was analyzed by t‐test. (^*^ *p* < 0.05, ^**^ *p* < 0.01, ^***^ *p* < 0.001, ^****^ *p* < 0.0001).

To verify the universality of M‐CP as a vaccine adjuvant, the effectiveness of M‐CP was further evaluated in other types of recombinant protein vaccines (herpes zoster vaccine) and more complex lysis vaccines (the influenza vaccine). Influenza is an acute respiratory infectious disease that annually causes 3 to 5 million new cases globally. Influenza vaccination is currently the most effective strategy to prevent seasonal influenza virus infection and reduce the mortality of influenza‐related diseases.^[^ ^14^ ^]^ At present, the adjuvant‐free influenza vaccine is one of the most commonly used forms. However, the rapid mutation of the influenza virus directly reduced the protective effect of the vaccine. Therefore, a compatible vaccine adjuvant that could offer the possibility to quickly respond to virus mutations and provide long‐lasting immune protection is in urgent need. In this study, tetravalent influenza lysis antigens (named Hemagg, containing influenza A virus H1N1 (A1) and H3N2 (A3), and influenza B virus Victoria (BV) and Yamagata (BY)) were used as the positive control. In addition, an aluminum adjuvant‐containing vaccine (named Hemagg‐Al) and the M‐CP peptide‐containing vaccine (named Hemagg‐M‐CP) were also prepared with Hemagg, and mice were immunized with a 14‐day interval (**Figure** **3**a).

Take the anti‐Yamagata hemagglutinin (anti‐BY) antibody for example, the results revealed that the Hemag group (four hemagglutinin antigens‐containing influenza lysis) only led to moderated anti‐BY antibody titer at day 28 (Figure 3b). Moreover, the introduction of aluminum adjuvant had no benefit on antibody response compared to the Hemagg group. In contrast, Hemagg‐M‐CP induced a 2.4‐fold higher geometric mean titer (GMT) than the Hemagg group, showing an adjuvant dose‐dependent response (Figure 3b). Simlar phenomena were also discovered in the detection of anti‐BY antibody titer at day 42 (Figure 3c), and anti‐A1 antibody titer at day 28 (Figure S4a, Supporting Information). These results indicated that M‐CP has the potential to be an effective adjuvant for more complex vaccines.

In addition, the ability of M‐CP to induce humoral immune responses to four antigens was also examined on day 82 after the first intramuscular injection. As shown in Figure 3d,f,g, significantly higher anit‐A1, anti‐BV, and anti‐BY antibodies were detected in mouse serum of the M‐CP‐containing group compared to an adjuvant‐free vaccine 10 weeks after the final immunization. Moreover, the anti‐A1 (Figure 3d), anit‐A3 (Figure 3e), and anti‐BY antibodies (Figure 3g) in the Hemagg‐2*M‐CP group resulted in the highest GMT, showing the outstanding application potential of stapling peptide adjuvant over aluminum adjuvant for compatibility with influenza vaccines. From the GMT statistical data, the M‐CP adjuvant also demonstrated an advantage in assisting the antibody response of B‐type influenza antigens. This phenomenon was different with Fluad Quadrivalent, the first MF‐59 adjuvant‐containing tetravalent influenza vaccine approved by the FDA in 2020.^[^ ^15^ ^]^ Therefore, we strongly speculate that M‐CP can be an alternative adjuvant for MF‐59 in the future. In addition to humoral immunity, the long‐term efficacy of M‐CP was also tested at the cellular immune level by re‐stimulating splenocytes with tetravalent influenza lysis antigens. The results displayed that restimulation led to 150% splenocyte proliferation in Hemagg‐M‐CP‐treated mice (Figure S4c, Supporting Information). Further antibody labeling staining data in Figures 3h,i, and S4d (Supporting Information) also showed the efficacy of M‐CP adjuvant in inducing central memory and effector memory T cell responses, which was consistent with the previous hepatitis B vaccine analysis. These results again demonstrated that M‐CP could not only induce effective humoral immunity but also activate cellular immunity, improving the long‐term efficacy of vaccines.

The compatibility of M‐CP was further revalidated in the herpes zoster vaccine (Figure 3a). Herpes zoster is an acute infectious skin disease caused by the reactivation of the varicella‐zoster virus (VZV), and no specific medication is currently available for neuralgia, which is one of the symptoms of this disease.^[^ ^16^ ^]^ At present, the vaccine containing the recombinant antigen glycoprotein E (gE) has been used to overcome the decline in immune function of elderly population.^[^ ^17^ ^]^ Thus, gE protein was mixed with M‐CP to obtain a novel zoster vaccine here. Our results revealed that M‐CP with gE protein not only elicited significant gE‐specific IgG antibody response, but also boosted 1.2,1.5, and 1.8‐fold higher in IgG1, IgG2a, and IgG 2b antibodies titers than the gE‐Al group (Figure 3j), respectively. Furthermore, compared to the aluminum adjuvant group, the re‐stimulation of splenocytes from the gE‐M‐CP treated mice showed more IFN‐γ^+^ immune spots (Figure 3k), indicating a stronger cellular immune response induced by the M‐CP‐containing vaccine. Taken together, we conclude that this sulfonium‐base peptide could be used as a generic adjuvant for multiple types of vaccines by boosting cellular immunity and humoral immunity.

### 2.4 Stapling Peptide Exerts Adjuvant Properties with the Help of Sulfonium Centers

The action mechanism of vaccines is still an important challenge in the field of vaccine research. Unlike the commonly used multi‐component adjuvants, M‐CP is chemically defined and has a single component, which provides convenience for the in‐depth analysis of its possible action mechanism as a vaccine adjuvant. Thus, on the one hand, the antigen delivery performance of M‐CP and its impact on downstream pathways were analyzed from a biological functional perspective. On the other hand, the key structural information of stable peptide adjuvants was deeply explored by chemical redesign and modification.

**Figure 4:**
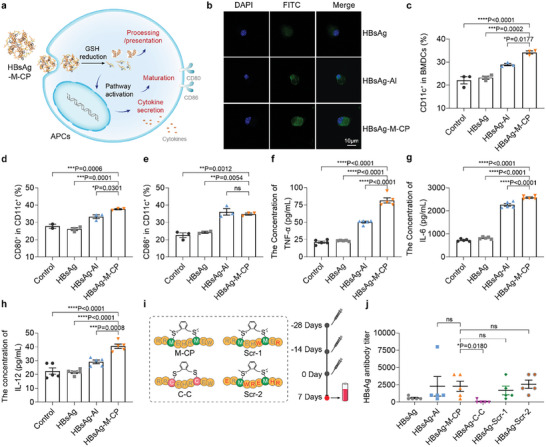
M‐CP promotes antigen uptake and APCs activation. a) The schematic diagram illustrated the possible mechanism of M‐CP adjuvant by inducing the cellular uptake of nano‐vaccine, DC cell maturation, and secretion of cytokines. b) Localization of internalized HBsAg in BMDCs after 4 h incubation with HBsAg, HBsAg‐Al, or HBsAg‐M‐CP. DAPI (blue) stained for the cell nuclei. FAM‐labeled HBsAg protein. Scale bar = 10 µm. c–e) Maturation of BMDCs after nano‐vaccine treatment for 24 h determined by the proportion of CD11c^+^, CD80^+^CD11c^+^, and CD86^+^CD11c^+^ cells. f–h) The concentrations of TNF‐α, IL‐6, and IL‐12 in the supernatant of BMDCs after 24 h incubation with different formulations. i) Diagram of the redesigned peptide adjuvant based on M‐CP. j) The anti‐HBsAg antibody titer of HBsAg protein‐redesigned adjuvant‐treated mice 1 week after the last immunization (n = 5). The data were presented as mean ± SEM (n ≥ 3) and analyzed by one‐way ANOVA with Turkey multiple comparisons post‐test (^*^ *p* < 0.05, ^**^ *p* < 0.01, ^***^ *p* < 0.001, ^****^ *p* < 0.0001).

Usually, adjuvants in vaccines act as carriers to achieve sustained release and efficient delivery of antigens.^[^ ^11^, ^18^ ^]^ Our previous studies have illustrated that sulfonium‐based stapling peptide is an excellent delivery system for nucleic acid drugs and neoantigen peptides.^[^ ^8^, ^10^, ^19^ ^]^ Therefore, we speculated that M‐CP with two positively charged sulfonium centers may exert its adjuvant properties by assisting antigen entry into APCs. To test this assumption, the indicated formulations were incubated with immature BMDCs (immature antigen‐presenting cells) and analyzed by laser confocal microscope. As shown in **Figure** **4**a,b, HBsAg protein could barely penetrate the cell membrane. With the help of an adjuvant, significant FAM fluorescence of anti‐HBsAg antibody was detected in BMDCs. In addition, BMDCs treated with HBsAg‐M‐CP displayed a stronger FAM signal than that of the HBsAg‐Al group, indicating the effective delivery of HBsAg antigen by M‐CP.

In addition, the maturation of BMDCs and secretion of cytokines were also measured. After 24 h incubation with the indicated formulations, all adjuvant‐containing groups induced higher expression of DC maturation biomarkers (CD86 and CD80 costimulatory molecules) compared with the control groups (Figure 4c–e). In particular, HBsAg‐M‐CP stimulated a more potent expression of CD80 in BMDCs. Similar results were also observed in the secretion of inflammatory factors in the HBsAg‐M‐CP treatment group. As shown in Figure 4f–h, HBsAg‐M‐CP induced the maximum responses of TNF‐α, IL‐6, and IL‐12, showing 1.6, 1.1, and 1.4 times higher than that of the HBsAg‐Al group, respectively. Our previous research indicated that M‐CP needs to synergistically induce immune activation with antigens at the cellular level, rather than by M‐CP alone.^[^ ^10^ ^]^ Moreover, as shown in Figure 4c–h, the synergistic effect of the M‐CP adjuvant is stronger than that of the Al adjuvant in inducing BMDCs maturation and promoting the secretion of cytokines. Taken together, we speculated that M‐CP exerts its adjuvant function by assisting antigen entry into antigen‐presenting cells (APCs) and further promoting the activation of downstream pathways. The enhanced maturation of DC and secretion of cytokines provided the possibility for the subsequent activation of B cells and T cells.

To further trace the adjuvant properties of the peptide, the M‐CP was chemically re‐designed. From the perspective of chemical structure (Figure S1, Supporting Information), the adjuvant properties of M‐CP can be attributed to cyclization, sulfonium center, and sequence. Considering that open‐loop design will directly lead to a decrease in delivery capacity,^[^ ^10^ ^]^ only the sequences arrangement and sulfonium centers were included in the scope of chemical structure redesign to control for single factor variables (Figures 4i and S5, Supporting Information). Previous studies have pointed out that microbial components, such as CpG and mono‐phosphoryl lipid A (MPL), were often included in the mixed adjuvants.^[^ ^20^ ^]^ Thus, we speculated that the adjuvant properties of M‐CP may be partially due to its sequence homology with microorganisms. By sequence alignment, we discovered that M‐CP was different from human proteins and only partially homologous to bacterial proteins. It was different from traditional truncated peptides adjuvant derived from pathogenic microorganisms or self‐adjuvanted protein, indicating that the adjuvant properties of M‐CP might be independent of the sequence. To verify this hypothesis, the sequence of M‐CP was disrupted and two M‐CP‐like adjuvants (named Scr‐1 and Scr‐2, Figures 4i and S5, Supporting Information) with random sequences were constructed. As shown in Figure 4j, almost equal antibody titer responses in M‐CP, aluminum, and M‐CP‐like adjuvant groups (Scr‐1 and Scr‐2) were detected on day 7 after the final immunization. These results further confirmed our prediction that the order of the amino acid sequences of M‐CP was not a decisive factor in its adjuvant properties. While, once the two sulfonium centers on M‐CP were removed, C‐C (Figure S5c, Supporting Information), with a similar delivery effect to M‐CP, could hardly induce an anti‐HBsAg antibody response in vivo (Figure 4j). These results demonstrated that the positively charged sulfonium centers play an important role in immune activation. Although M‐CP remains the most promising candidate for peptide adjuvants in this study, our results well indicated the modifiability of this peptide‐based adjuvant. Predictably, this discovery of sulfonium in immune enhancement will provide a huge space for vaccine adjuvant design and antigen modification. Of course, more studies on the design of peptide‐based adjuvant and in‐deep mechanism analysis of sulfonium are underway.

### 2.5 Stapling Peptide Exhibits Good Biosafety as a Vaccine Adjuvant Candidate

**Figure 5:**
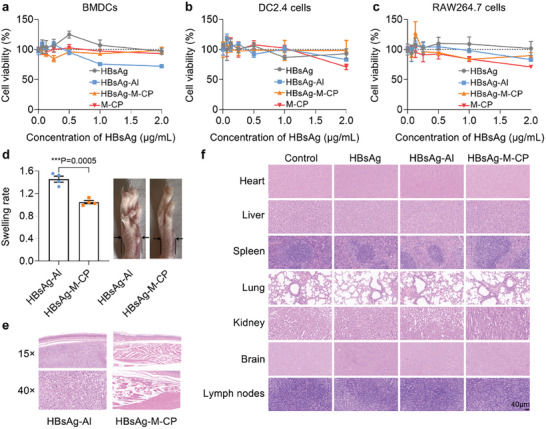
M‐CP shows good biosafety in vitro and in vivo. a–c) Cytotoxicity assessment of HBsAg‐Al and HBsAg‐M‐CP (calculated based on HBsAg concentration) to a) BMDCs, b) DC2.4, and c) RAW264.7 cells. d) Swelling factor of footpads (n = 4) and images of soles at day 8 after footpad injection with nano‐vaccine. The data were presented as mean ± SEM and analyzed by t‐test (^***^ *p* < 0.001). e) H&E‐stained paw sections of mice at day 8. f) H&E‐stained main organ sections collected from the HBsAg containing vaccines treated mice at week 6. Scale bar = 40 µm.

Vaccines are the most effective and widely used tool for viral infection prevention.^[^ ^21^ ^]^ The safety of adjuvants is one of the decisive factors for their clinical application. Here, the bio‐compatibility and safety of M‐CP are important parts of verifying its vaccine application. Here, the safety of M‐CP was evaluated in vitro and in vivo. As shown in **Figure** **5**a, neither M‐CP nor HBsAg‐M‐CP affected the proliferation of BMDCs cells at 150 µm (M‐CP concentration) after 24 h incubation. The same phenomena were observed in the DC2.4 and RAW264.7 cells (Figure 5b,c). In addition, unlike the vast amount of aluminum adjuvant adhering to the BMDCs membrane (Figure S6a, Supporting Information), the HBsAg‐M‐CP treatment group had almost no effect on cell morphology, which was similar to the control group.

Furthermore, the biocompatibility of M‐CP in mice was also evaluated. The footpad injection experiment was carried out to characterize the acute inflammatory responses raised by the adjuvant. The results displayed that M‐CP triggered a weak local inflammatory response, showing insignificant changes in footpad color and thickness (Figure 5d). In contrast, the aluminum‐containing vaccine‐immunized group caused obvious swelling of footpads, with a thickness increase of 140% at 8 days (Figure 5d). In addition, the images of H&E‐stained sections also support this phenomenon. As shown in Figure 5e, more immune cells were recruited in the injection site in the HBsAg‐Al‐immunized group. This might happen due to the stronger tissue retention of aluminum, which was consistent with the morphological observations at the cellular level mentioned above. Previous studies have also stated that patients vaccinated with aluminum‐containing vaccines may occasionally experience adverse reactions such as granulation and redness.^[^ ^4^ ^]^ In the group of HBsAg‐M‐CP treated mice, no significant histological changes were observed in the footpads at 8 days. Therefore, we speculated that the significant reduction of the local acute inflammatory response induced by peptide‐containing vaccines will further minimize the side effects of the vaccine and improve patient compliance.

In addition, the blood on week 2 after the last immunizations was also collected and tested as the clinical signs of the safety of the HBsAg‐M‐CP vaccine. As shown in Table S1 (Supporting Information), compared with the control and HBsAg groups, there was no significant change in the relevant hematological indicators of the HBsAg‐M‐CP group, showing the good safety of HBsAg‐M‐CP vaccine. To evaluate the long‐term safety of stapling peptide adjuvant, the immunogenicity of M‐CP and the H&E staining of major organs at 6 weeks after three doses of intramuscular injection were also analyzed. The antibodies against the M‐CP or M‐LP in serum were tested by ELISA. As shown in Figure S6b,c (Supporting Information), there was no significant difference in anti‐M‐CP or anti‐M‐LP antibody response between HBsAg and HBsAg‐M‐CP treatment groups. The results indicated that M‐CP cannot induce autoimmunity signals within 6 weeks, indicating the good safety of stapling peptide as a novel vaccine adjuvant. Similar results can also be observed from the H&E staining results. As shown in Figure 5f, no obvious signs of inflammatory lesions and organ damage were observed in the major organs of all M‐CP treatment groups. Taken together, these above results demonstrated that M‐CP could be used as a bio‐safe adjuvant and hold great potential for applications in human and veterinary vaccines.

## 3 Conclusion

In summary, we have reported for the first time a general and effective peptide adjuvant candidate with only 9 amino acids based on the sulfonium stapling peptide strategy. This peptide adjuvant with a chemically defined structure has not only shown to be suitable for recombinant protein vaccines but is also compatible with split vaccines. Different from the traditional aluminum adjuvants, peptide adjuvants could induce simultaneous activation of humoral and cellular immunity by assisting antigens entry into APCs, and hold great potential for developing therapeutic vaccines. It should be noted that no unnatural amino acids were needed for the preparation of this peptide adjuvant, which directly reduces the production cost of the vaccines. Considering the definite stereo‐structure, quick and commercialized preparation process, and excellent bio‐safety of peptides, our stapling peptide adjuvant will offer a candidate for improving outcomes and long‐term effectiveness of vaccines in clinical research. Moreover, the functionalization and improvement of peptide adjuvants can be easily achieved by chemically changing the amino acids and ring types or introducing new modified groups. Predictably, the integration of AI‐assisted design and peptide structure optimization will provide a new paradigm for the rational design of adjuvants and the construction of an adjuvant library. In the future, this programmable peptide‐based adjuvant is expected to meet the needs of multiple vaccines (such as mRNA, peptide, and protein vaccines).

## 4 Experimental Section

### Materials and Reagents

All peptide adjuvants used in this research include M‐CP (Met‐closed peptide with two sulfonium centers, Fmoc‐RRMEHRMEW), Scr‐1 (Met‐closed scramble peptide 1 with two sulfonium centers, Fmoc‐RRMEHWMER), Scr‐2 (Met‐closed scramble peptide 2 with two sulfonium centers, Fmoc‐ERMWREMHR), and C‐C (Cys‐closed peptide without sulfonium center, Fmoc‐RRCEHRCEW). The recombinant HBsAg protein, tetravalent influenza lysis antigens, recombinant antigen glycoprotein E (gE) of varicella‐zoster virus, and corresponding antibody detection ELISA kits were from Shenzhen Kangtai Biological Products Co., Ltd. (Shenzhen, China). DAPI, flow cytometry staining buffer, and anti‐mouse antibodies (CD16/CD32 (clone 93), CD3e (eFluor450, clone 145‐2C11), CD4 (APC, clone RM4‐5), CD8a (FITC, clone 53–6.7), CD19 (PE, clone 6OMP31), CD44 (PE, clone IM7), CD62L (APC, clone MEL‐14), CD11c (APC, clone N418), CD80 (FITC, clone 16‐10A1), CD86 (PE, clone GL1)) for flow cytometry staining were purchased from Thermo Scientific (Pittsburgh, PA, USA). 7‐AAD for identifying live and dead cells was purchased from Biolegend (San Diego, USA). IL‐4 and GM‐CSF for cell culture of BMDCs were purchased from MedChemExpress (New Jersey, USA). Cytokines (TNF‐α, IL‐12, and IL‐6) detection kits were purchased from Elabscience Biotechnology Co. Ltd. (Wuhan, China). IFN‐γ ELISpot kit was purchased from MABTECH (Sweden). 10 × lysing buffer for the preparation of splenic single cells was purchased from BD (USA). Other chemical materials were purchased from Sigma‐Aldrich (St. Louis, MO, USA).

### Animals and Cell Lines

In this research, 6–8 weeks Balb/c mice and C57BL/6 (female) were purchased from Guangdong Vital River Laboratory Animal Technology Co., Ltd. (Guangdong, China). All animal‐related experiments complied with the “The Guide for the Care and Use of Experimental Animals” published by the National Institutes of Health and approved by the Animal Care and Use Committee of Peking University Shenzhen Graduate School (SYXK (Guangdong) 2017‐0172, Shenzhen, China). RAW264.7 cell was purchased from ATCC and the DC2.4 cell was a gift from Prof. Xingjie Liang at the National Center for Nanoscience and Technology (Beijing, China). Myeloid‐derived dendritic cells (BMDCs) were isolated from the bone marrow of C57BL/6 mice and induced by IL‐4/GM‐ CSF‐containing culture medium as described in the previous study.^[^ ^10^ ^]^ All cells were cultured in RPMI1640 complete medium (containing 10% fetal bovine serum, 100 µg mL^−1^ streptomycin, and 100 U mL^−1^ penicillin) at 37 °C in 5% CO_2_. No mycoplasma or rodent pathogen infection was detected.

### Synthesis and Modification of Peptide Adjuvants

The linear peptides used in this study were synthesized on rink amide‐resin by the standard solid‐phase peptide synthesis strategy and were cyclized as described in the previous report.^[^ ^10^ ^]^ In detail, for the preparation of M‐CP, Scr‐1, and Scr‐2, the freeze‐dried products of linear peptides were mixed with 5 eq of α, α’‐dibromo‐o‐xylene in 10% formic acid buffer (acetonitrile: formic acid: water (V/V/V) = 5:1:4), followed by overnight reaction at room temperature on a shaker. In contrast, the linear peptide product of C‐C reacted with α, α’‐dibromo‐o‐xylene in an alkaline condition (acetonitrile: DIPEA: water (V/V/V) = 5:1:4) for 6 h and purified by HPLC. Then the molecular weight of peptide products was identified by LC‐MS.

### Characterization and Optimization of Nano‐Vaccine

To evaluate the optimal addition amount of peptide adjuvant, different concentrations of M‐CP (62.5, 125, 250, 500, and 1000 µg) were mixed with 2 µg HBsAg protein in 1 mL PBS buffer at room temperature for 30 min. Then the particle size and zeta potential were analyzed by dynamic light scattering (DLS, Malvern Zetasizer, Britain). This concentration of M‐CP which hardly affects the negative charge of HBsAg was chosen for subsequent research. In addition, DLS was also used to evaluate the stability of nano‐vaccine after incubation in PBS buffer at 4 °C for up to 105 days. To observe the micromorphology of nano‐vaccine, the mixture was dropped onto thin carbon‐coated grids and negative stained with 1% uranyl acetate. After blotting and air‐drying, the carbon‐coated grid was observed by scanning electron microscope (TEM, FEI Tecnia G2 spirit, Netherlands).

### Animal Immunization and Antibody Titer Determination

To evaluate the effectiveness of peptide adjuvant, 6–8 weeks Balb/c mice were immunized with 100 µL indicated formulations by intramuscular injection at the left thigh. In the test of the hepatitis B vaccine (n = 6), M‐CP (250 µg) was mixed with recombinant HBsAg protein (2 µg) in PBS buffer and mice were immunized three times in 14‐day intervals. Here, a commercial hepatitis B vaccine with aluminum adjuvant (2 µg HBsAg protein) was used as the positive control.^[^ ^10^ ^]^ The serums of immunized mice were collected at 1, 2, 4, and 6 weeks after the last immunization and analyzed by ELISA kits according to manufacturing instructions. In the test of the optimal dosage of peptide adjuvant (n = 6), different doses of M‐CP (0, 62.5, 125, and 250 µg) were mixed with recombinant HBsAg protein (2 µg) and the mice were immunized three times in 14‐day intervals. The serums were collected and tested 2 weeks after the last immunization. In the test of influenza vaccine (n = 8), Balb/c mice were treated with nano‐vaccine containing influenza A virus H1N1 lysis (1.5 µg), influenza A virus H3N2 lysis (1.5 µg), influenza B virus Victoria lysis (1.5 µg), influenza B virus Yamagata lysis (1.5 µg), and M‐CP (250 µg) two times in 14‐day intervals. The serums were collected on days 28, 42, and 82. The mixture of aluminum adjuvant (250 µg) and 6.0 µg tetravalent influenza vaccine was used as the positive control. In the test of herpes zoster vaccine (n = 8), Balb/c mice were vaccinated with nano‐vaccine (250 µg M‐CP adjuvant and 5 µg recombinant gE protein per mouse) at day 0 and day 21. The blood was harvested on day 28, followed by an analysis of anti‐gE antibody titer. The mixture of aluminum adjuvant (50 µg) and gE protein was used as the positive control.

### Phenotypic Assessment of Immunocytes in PBMCs and Spleen

The humoral immunity and cellular immunity responses induced by M‐CP adjuvant were also evaluated through changes in the proportion of B and T cells in PBMCs. In detail, the blood of Balb/c mice was collected at week 6 after the last immunization and lysed with RBC lysis buffer to isolate PBMCs. Then PBMCs were stained with CD16/CD32 (1 µL) at room temperature for 10 min and an antibody cocktail containing CD3e (eFluor450), CD19 (PE), CD4 (APC), and CD8a (FITC) antibodies for 20 min. After washed with PBS, the cells were analyzed by flow cytometry. To assess the immunologic memory and CTL cytotoxicity of lymphocytes, spleens at the end of animal experiments were harvested and ground into a single‐cell suspension. Take the HBsAg vaccine immunization group for example, splenocytes were re‐stimulated with 5 µg mL^−1^ HBsAg protein for 60 h, stained with the marker antibodies, and analyzed by flow cytometer to distinguish the central memory T cells (CD44^+^CD62L^+^CD3^+^ T cells) and effector memory T cells (CD44^+^CD62L^−^CD3^+^ T cells). The re‐proliferation ability of lymphocytes was also evaluated by CCK8 assays after incubating with 5 µg mL^−1^ HBsAg protein for 60 h. Similar to the hepatitis B vaccine group, the influenza‐treated group at day 82 also underwent the same evaluation processes. In the test of herpes zoster vaccine, the ELISPOT assay of IFN‐γ was performed on day 28 following the manufacturer's instructions to assess the CTL cytotoxicity of lymphocytes.

### Intracellular Localization of Nano‐Vaccine in BMDCs

Here, the internalized HBsAg proteins in BMDCs were labeled through an immunofluorescence strategy and imaged by a confocal laser scanning microscopy (Nikon A1R, Japan). Briefly, 1 × 10^5^ cells were plated in 24‐well plates and incubated with free HBsAg, HBsAg‐Al, and HBsAg‐M‐CP (2 µg HBsAg protein, and 250 µg M‐CP) at 37 °C for 4 h, respectively. After washing three times with PBS, the cells were fixed with 4% formaldehyde at 4 °C for 15 min and washed three times again with PBS. Later, the cells were sequentially treated with 5% milk at room temperature for 15 min, anti‐HBsAg mouse antibody at 4 °C for 12 h, and FITC‐conjugated anti‐mouse IgG antibody at room temperature for 15 min. After washing three times with TBST at 5 min intervals, the cells were sealed on the microscopic slides with a DAPI‐containing sealing agent and imaged with confocal laser scanning microscopy.

### The Maturity of BMDCs and Secretion of Cytokines

1 × 10^6^ BMDCs were plated in 24‐well plates and incubated with free HBsAg, HBsAg‐Al, and HBsAg‐M‐CP (2 µg HBsAg protein and 250 µg M‐CP) at 37 °C for 24 h, respectively. Then the cells were harvested and stained with CD16/CD32 (1 µL) at room temperature for 10 min and an antibody cocktail containing CD11c (APC), CD80 (FITC), and CD86 (PE). After 20 min incubation, the cells were washed with PBS and analyzed by flow cytometry to evaluate the maturity of BMDCs induced by nano‐vaccine. In addition, the cell culture supernatant was collected and analyzed by TNF‐α, IL‐6, and IL‐12 ELISA kits following the manufacturer's instructions.

### Safety Evaluation In Vitro and In Vivo

CCK8 test was carried out in vitro to estimate the safety of nano‐vaccine. Take BMDCs for example, 8000 BMDCs/well were plated in 96‐well plates. After 24 h treatment with different concentrations of nano‐vaccine, each well was added with 10 µL CCK8 solution and the plate was incubated at 37 °C for another 4 h. The absorbance of formazan was measured at 450 nm by a microplate reader (BioTek H1, USA). Unless otherwise specially declared, the cytotoxicity of nano‐vaccine to DC2.4 and RAW264.7 cells was determined using the same method. In vivo, the safety of the nano‐vaccine was evaluated by inflammation response at the injection site. After injection of 30 µL HBsAg‐Al or HBsAg‐M‐CP at the footpad, the thickness of the footpad was measured at day 0 and day 8. Swelling factor = (thickness_day8_‐thickness_day0_)/thickness_day0_. The recruitment of immune cells in soles was analyzed by the H&E staining experiment. In addition, to estimate the medium‐ and long‐term safety of nano‐vaccine, the blood and serum at week 2 and week 6 were collected. The blood at 2 week was analyzed by flow cytometry to obtain hematological data. The antibodies against the M‐CP or M‐LP in serum at week 6 were tested by ELISA as described in previous report. The main organs in the HBsAg immunization group at week 6 were also harvested and stained with H&E.

### Statistical Analysis

All data were presented as mean ± SEM from at least three independent experiments (n ≥ 3) and analyzed by Prism 8 (GraphPad Software). Student's t‐test and one‐way ANOVA with Turkey multiple comparisons post‐test were selected for comparing differences between groups. Unless otherwise indicated, the comparison was considered statistically significant if *p* < 0.05.

## Conflict of Interest

The authors declare no conflict of interest.

## Author Contributions

Y.P.Z., C.S.L., and W.L.L. contributed equally to this work. Y.P.Z., C.S.L., and W.L.L. designed research and provided the necessary bioinformatics analysis for this study. L.Y.J., H.T.L., J.L., and X.M.Z. provided the necessary help for the animal experiment in this study. Y.P.Z. and Y.X. wrote the original draft. J.H.G. participated in the experimental design and discussion. Conceptualization, reviewing, and supervision by Z.G.L. and F.Y.

## Supporting information



Supporting Information

Supporting Information

## Data Availability

The data that support the findings of this study are available in the supplementary information of this article.
