# Supplementary material for: Programmable Stapling Peptide Based on Sulfonium as Universal Vaccine Adjuvants for Multiple Types of Vaccines
Source: Adv Sci (Weinh). 2025 Jan 29;12(11):2409567. doi: 10.1002/advs.202409567 (PMC11923873; doi:10.1002/advs.202409567)
Supplement: Supplementary file 1 — Supporting Information [file ADVS-12-2409567-s001.docx]

Supporting Information

**Programmable stapling peptide based on sulfonium as universal vaccine adjuvants for multiple types of vaccines**

Yaping Zhang, Chenshan Lian, Wenlong Lai, Leying Jiang, Yun Xing, Huiting Liang, Jin Li, Xinming Zhang, Jianhui Gan*, Zigang Li*, and Feng Yin*

Y.P. Zhang, C.S. Lian, L.Y. Jiang, Y. Xing, Z.G. Li, F. Yin

State Key Laboratory of Chemical Oncogenomics, School of Chemical Biology and Biotechnology, Peking University Shenzhen Graduate School, Shenzhen 518055, P.R. China

E-mail: lizg@pkusz.edu.cn; yinfeng@szbl.ac.cn

Y.P. Zhang, H.T. Liang, Z.G. Li, F. Yin

Pingshan Translational Medicine Center, Shenzhen Bay Laboratory, Shenzhen 518118, P.R. China

W.L. Lai, J. Li, J.H. Gan

Shenzhen Kangtai Biological Products Co. Ltd., Shenzhen 518057, P.R. China

E-mail: ganjh@biokangtai.com

X.M. Zhang

Beijing Minhai Biotechnology Co. Ltd., Beijing 102609, P.R. China

Keywords: stapling peptides, sulfonium center, nano-vaccine, universal adjuvant.


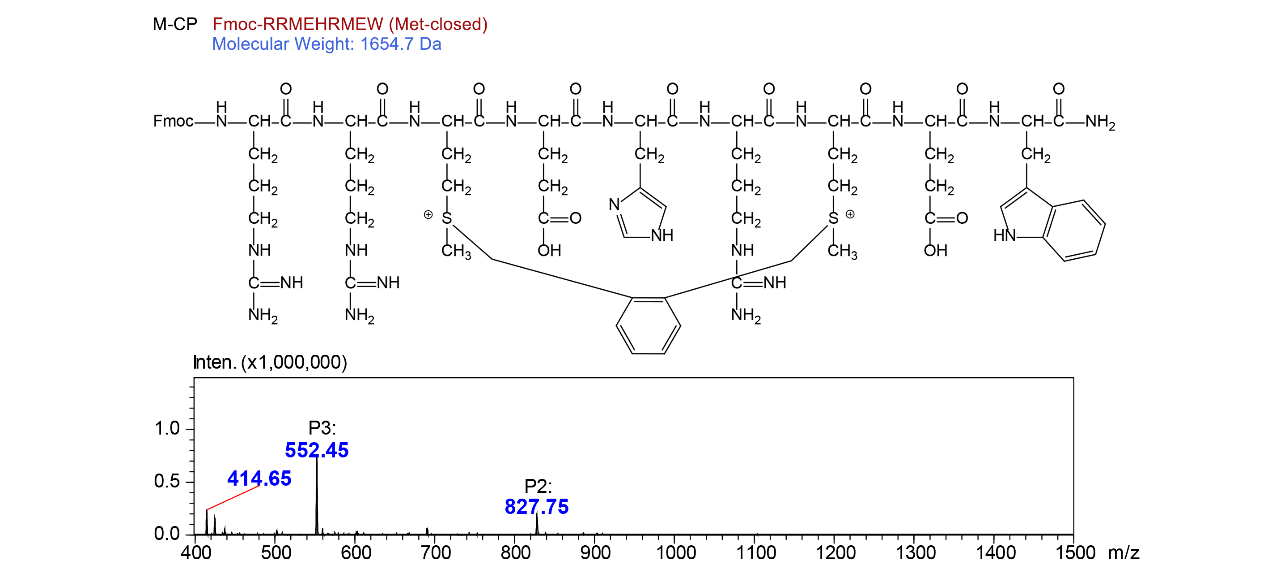


**Figure S1.** The chemical structure and MS result of M-CP (Met-closed peptide with two sulfonium centers).


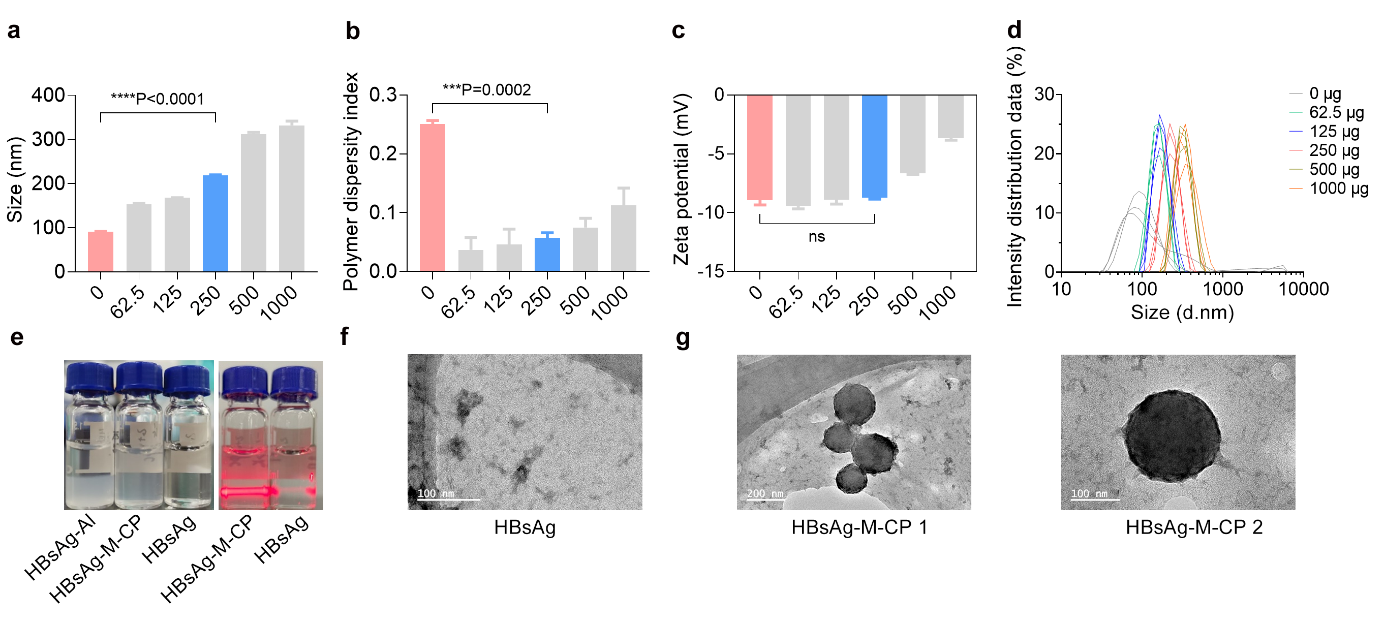


**Figure S2.** Characterization of nano-vaccines. a-c) The a) average particle size, b) polymer dispersity index (PDI), c) zeta potential, and d) particle intensity distribution curves of nano-vaccine under different M-CP concentrations analyzed by dynamic light scattering. Different concentrations of M-CP (0, 62.5, 125, 250, 500, and 1000 μg) were mixed with 2 μg HBsAg protein in 1 mL PBS buffer at room temperature for 30 min. e) Tyndall phenomenon of vaccine solution. [HBsAg] = 2 μg mL^-1^, [M-CP] = 250 μg mL^-1^. f,g) Transmission electron microscope images of f) HBsAg and g) HBsAg-M-CP. Scale bar = 200 nm. [HBsAg] = 2 μg mL^-1^, [M-CP] = 250 μg mL^-1^. The data were presented as mean ± SEM (n = 3) from three independent experiments. Data were analyzed by one-way ANOVA with Turkey multiple comparisons post-test (**P*<0.05, ***P*<0.01, ****P*<0.001, *****P*<0.0001).


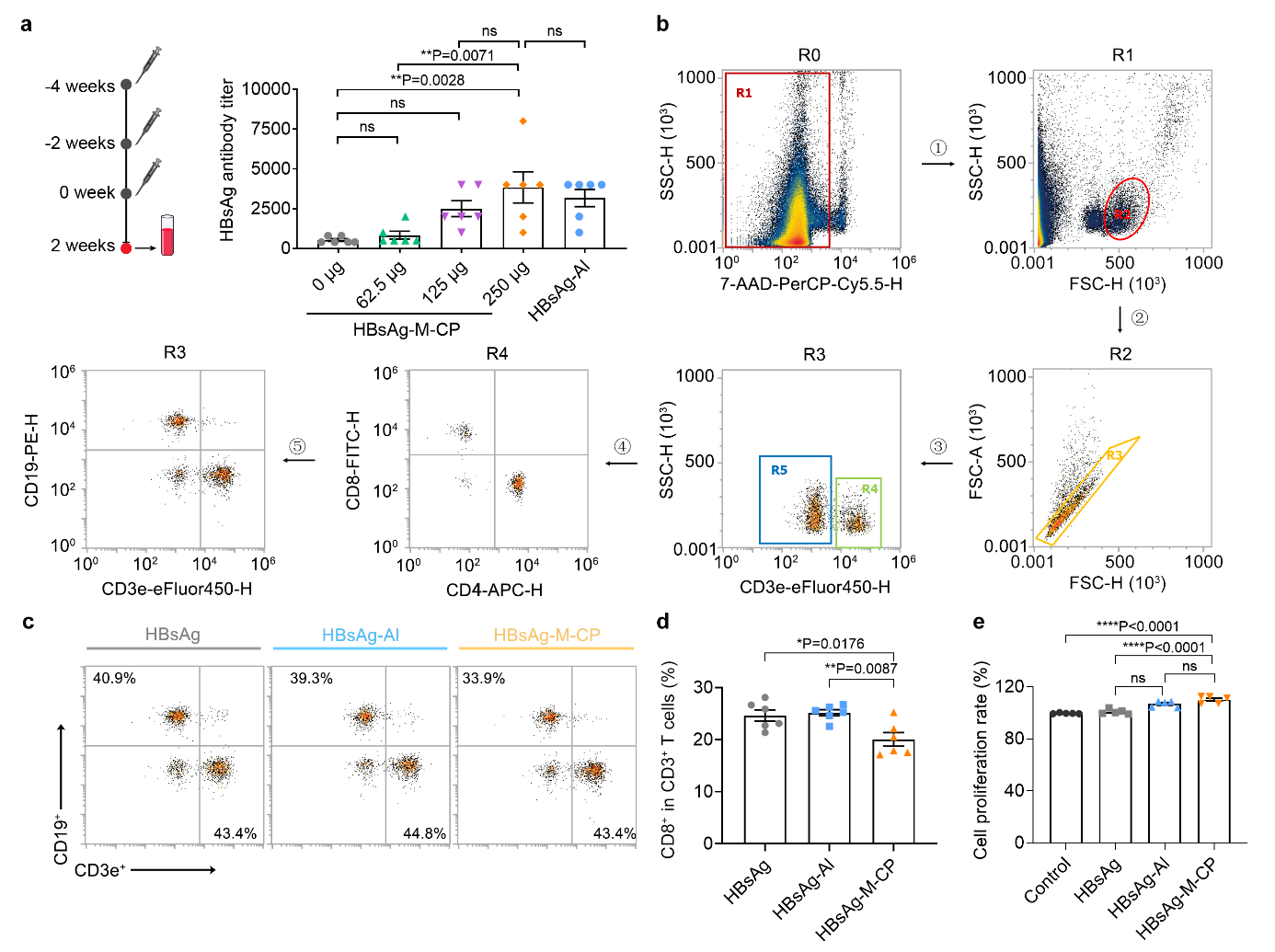


**Figure S3.** HBsAg-M-CP nano-vaccine induces the activation of the immune system *in vivo*. a) The anti-HBsAg antibody titer 2 weeks after the last immunization with different concentrations of M-CP adjuvant (n=6). b) Gating strategy to evaluate the percentage of B cells (CD19^+^) and T cells (CD3e^+^) in peripheral blood at week 6 post-immunization after three immunizations. c) The representative scatter plots of CD19^+^ B-cells and CD3e^+^ T cells in peripheral blood at week 6. d) The percentage of CD8^+^ T cells in CD3e^+^ T cells at week 6 (n=6). e) Repopulation rate of immunocytes in the spleen after re-stimulation with HBsAg protein at week 6 post-immunization (n=5). The data were presented as mean ± SEM. Data were analyzed by one-way ANOVA with Turkey multiple comparisons post-test (**P*<0.05, ***P*<0.01, ****P*<0.001, *****P*<0.0001).


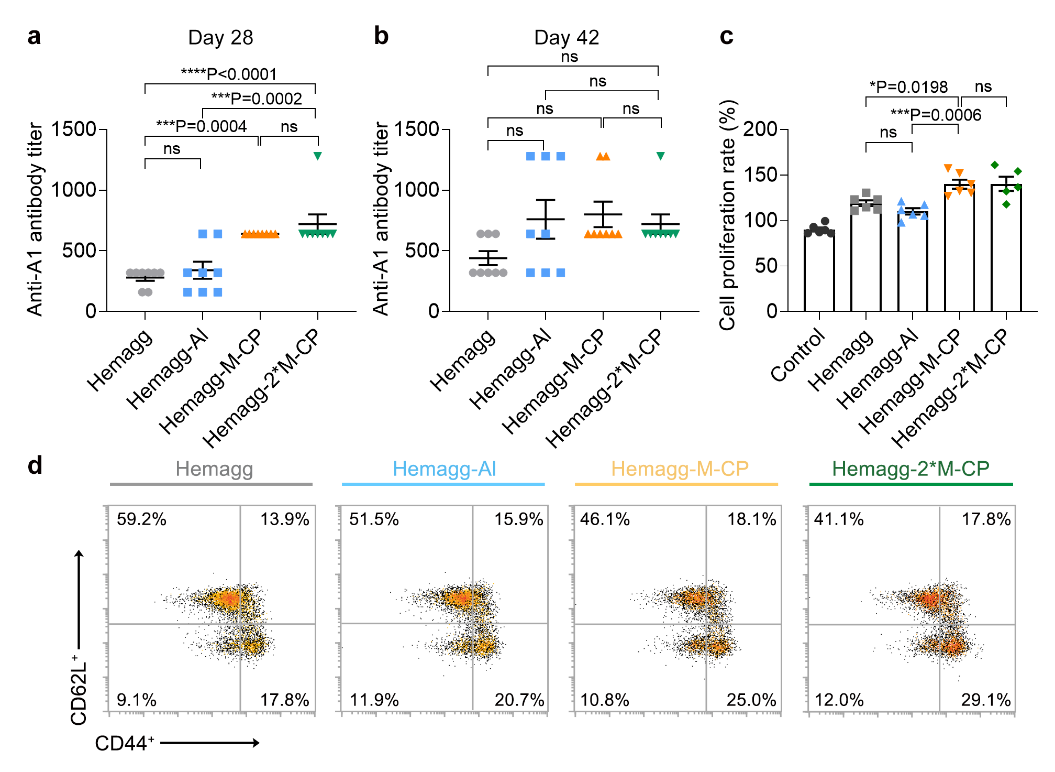


**Figure S4.** M-CP mixed with influenza vaccines induce effective antibody response and cellular immune response *in vivo*. a,b) The anti-H1N1 hemagglutinin antibody titer at a) day 28 and b) day 42 (n=8). c) Repopulation rate of immunocytes in the spleen after re-stimulation with influenza vaccines at day 82 post-immunization (n=6). d) The representative scatter plots of memory T cells in splenic single cells after re-stimulation with influenza vaccines. The data were presented as mean ± SEM. Data were analyzed by one-way ANOVA with Turkey multiple comparisons post-test (**P*<0.05, ***P*<0.01, ****P*<0.001, *****P*<0.0001).


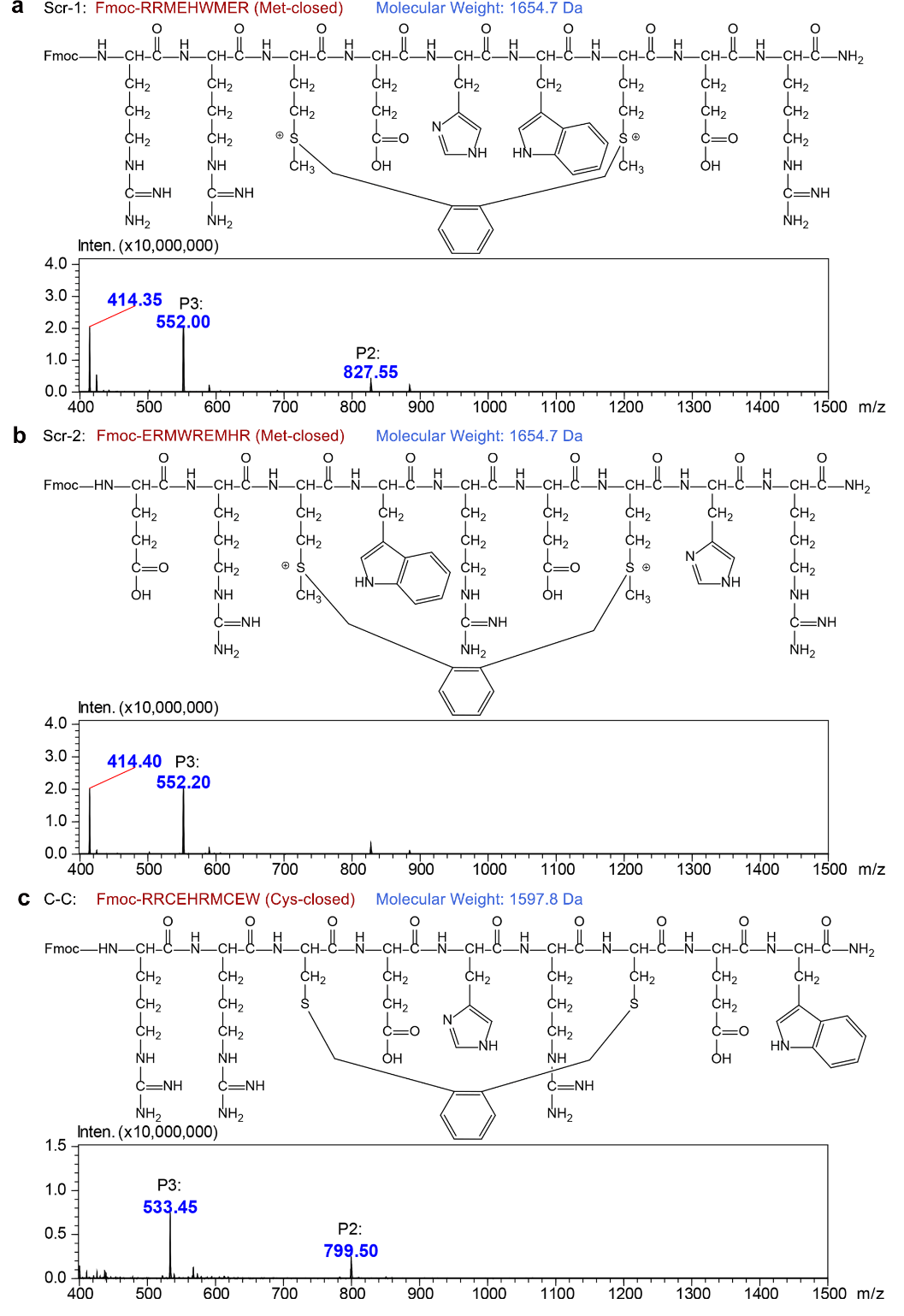


**Figure S5.** The chemical structures and MS results of the redesigned peptide adjuvant. a) Scr-1 peptide adjuvant (Met-closed scramble peptide 1 with two sulfonium centers). b) Scr-2 peptide adjuvant (Met-closed scramble peptide 2 with two sulfonium centers). c) C-C peptide adjuvant (Cys-closed peptide without sulfonium center).


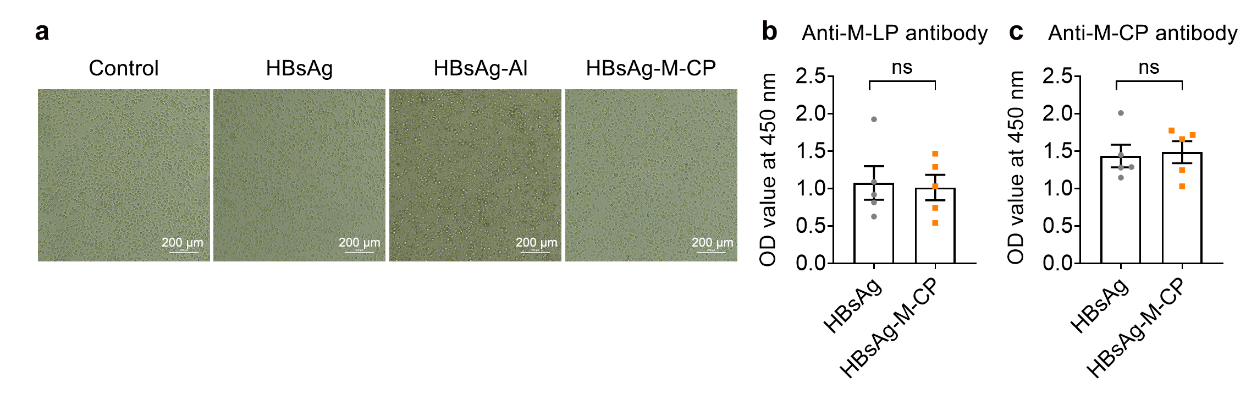


**Figure S6.** The safety evaluation of M-CP peptide adjuvant at the cellular and animal levels. a) Cell morphology after treatment with 1640 cell culture (Control), HBsAg protein, HBsAg-Al, and HBsAg-M-CP nano-vaccine for 24 h. Scale bar = 200 μm. b) the anti-M-LP or c) anti-M-CP antibodies in the HBsAg and HBsAg-M-CP treatment groups. Serums of mice at week 6 after three immunizations with HBsAg-M-CP (2 μg HBsAg and 250 μg M-CP) (n = 5) were analyzed by ELISA. The data were presented as mean ± SEM. Before conducting a significant difference analysis, the normality of data was analyzed by the “Normality and Lognormality Tests” option. Then, data were analyzed by parametric t-test with a two-tailed P value (ns: no significant difference).

**Table S1.** The hematology analysis on week 2 after the last immunizations.

| Testing indicators | Control  (*mean±SD*) | HBsAg  (*mean±SD*) | HBsAg-Al  (*mean±SD*) | HBsAg-M-CP  (*mean±SD*) |
| --- | --- | --- | --- | --- |
| WNC (10^9^/L) | 5.72±0.55 | 6.24±1.32 | 4.52±1.14 | 5.00±0.54 |
| NEUT # (10^9^/L) | 0.83±0.05 | 0.88±0.22 | 0.86±0.13 | 0.60±0.15 |
| LYMPH # (10^9^/L) | 4.72±0.49 | 5.18±1.08 | 3.47±1.05 | 4.26±0.48 |
| MONO # (10^9^/L) | 0.11±0.02 | 0.12±0.04 | 0.13±0.03 | 0.08±0.01 |
| EO # (10^9^/L) | 0.06±0.04 | 0.07±0.02 | 0.07±0.04 | 0.07±0.04 |
| BASO # (10^9^/L) | 0 | 0 | 0 | 0 |
| NEUT % (%) | 14.50±0.70 | 14.00±0.67 | 19.90±5.60 | 11.85±2.49* |
| LYMPH % (%) | 82.60±0.77 | 82.95±0.49 | 75.75±5.42* | 85.13±1.85** |
| MONO % (%) | 1.85±0.29 | 1.95±0.52 | 2.78±0.05** | 1.65±0.26** |
| EO % (%) | 1.05±0.65 | 1.13±0.21 | 1.58±0.57 | 1.38±0.90 |
| BASO % (%) | 0 | 0 | 0 | 0 |
| RBC (10^12^/L) | 10.69±0.42 | 10.68±0.15 | 9.52±1.83 | 10.39±0.09 |
| HGB (g/L) | 171.00±6.06 | 171.50±0.58 | 152.00±35.52 | 168.25±2.06 |
| HCT (%) | 48.68±1.50 | 48.93±0.66 | 42.60±8.93 | 47.58±0.28 |
| MCV (fL) | 45.55±0.48 | 45.80±0.36 | 44.60±1.01 | 45.83±0.32 |
| MCH (pg) | 15.98±0.29 | 16.08±0.26 | 15.85±0.90 | 16.20±0.00 |
| MCHC (g/L) | 351.00±3.46 | 351.25±4.27 | 355.00±12.03 | 353.50±2.65 |
| RDW-CV (%) | 14.63±0.21 | 14.58±0.22 | 14.65±0.47 | 14.75±0.24 |
| RDW-SD (%) | 30.63±0.51 | 30.50±0.32 | 29.90±0.53 | 31.05±0.56* |
| PLT (10^9^/L) | 884.25±185.64 | 898.25±75.20 | 793.25±223.60 | 952.25±74.40 |
| MPV (fL) | 5.73±0.38 | 5.78±0.26 | 6.08±0.45 | 5.68±0.25 |
| PDW (fL) | 16.08±0.48 | 16.25±0.24 | 16.38±0.17 | 16.18±0.24 |
| PCT (%) | 0.50±0.09 | 0.52±0.03 | 0.47±0.11 | 0.54±0.03 |

Notes: The data were presented as mean ± SD (n = 4). Data were analyzed by one-way ANOVA with Turkey multiple comparisons post-test (**P*<0.05, ***P*<0.01, ****P*<0.001, *****P*<0.0001). The significant differences between the experimental group and the control group were marked with black asterisks. The significant differences between the HBsAg-Al treatment group and the HBsAg-M-CP treatment group were marked with red asterisks. WNC: White blood cell count; NEUT: neutrophil count/ratio; LYMPH: Lymphocyte count/ratio; MONO: Monocyte count/ratio; Eo: Eosinophil count/ratio; BASO: basophil count/ratio; RBC: Red blood cell count; HGB: Hemoglobin; HCT: Hematocrit; MCV: Mean corpuscular count; MCH: Mean corpuscular hemoglobin; MCHC: Mean corpuscular hemoglobin concentration; RDW-CV: Coefficient variation of red blood cell volume distribution width; RDW-SD: Standard deviation in red cell distribution width; PLT: Platelet; MPV: Mean platelet volume; PDW: Platelet distribution width; PCT: Thrombocytocrit.
